# Unbiased enrichment of urine exfoliated cells on nanostructured substrates for sensitive detection of urothelial tumor cells

**DOI:** 10.1002/cam4.2655

**Published:** 2019-11-10

**Authors:** Xin Wang, Yuanyuan Gu, Shiwei Zhang, Gangqiang Li, Tianyao Liu, Tianwei Wang, Haixiang Qin, Bo Jiang, Lin Zhu, Yajun Li, Haozhi Lei, Ming Li, Qun Zhang, Rong Yang, Feng Fang, Hongqian Guo

**Affiliations:** ^1^ Department of Urology Drum Tower Hospital Medical School of Nanjing University Institute of Urology Nanjing University Nanjing China; ^2^ PerMed Biomedicine Institute Shanghai China; ^3^ Department of Pathology Naval Characteristic Medical Center Shanghai China; ^4^ Department of Pathology The Affiliated Suzhou Municipal Hospital of Nanjing Medical University Suzhou China; ^5^ Department of Pharmacology Nanjing Medical University Nanjing China

**Keywords:** cytology, immunocytology, nanostructured substrates, urinary tumor cells, urothelial carcinoma

## Abstract

**Background:**

Early detection of urothelial carcinoma (UC) by noninvasive diagnostic methods with high accuracy is still underscored. This study aimed to develop a noninvasive assay incorporating both enrichment of urine exfoliated cells and immunoassays for UC detection.

**Methods:**

Polystyrene dishes were exposed to oxygen plasma and modified with 3‐aminopropyltriethoxysilane to prepare amine‐functionalized nanostructured substrates (NS). Performance characterization of NS was evaluated by atomic force microscope and X‐ray photoelectron spectroscopy. Urine exfoliated cells were captured by NS and then immunostained to detect urinary tumor cells (UTCs), which was called UTC assay. The receiver operating characteristic (ROC) curve, area under ROC curve (AUC), and Youden index were used to find the cutoff value of UTC assay. ROC analysis and McNemar test were used to compare the diagnostic accuracy of UTC assay with cytology. Kappa test was used to analyze the agreement of UTC assay and cytology with pathological diagnosis.

**Results:**

Nanostructured substrates had good cell binding yields of nucleated cells and tumor cells. CK20^+^CD45^−^CD11b^−^ cells were considered as UTCs. UTC number ≥ 1 per sample could be considered as a positive result. By AUC and Kappa analysis, UTC assay showed good performance in UC detection. McNemar test demonstrated that UTC assay had a superior sensitivity even in low‐grade subgroup and a similar specificity compared to cytology in UC diagnosis.

**Conclusions:**

Nanostructured substrates could be used to enrich the exfoliated cells from urine samples. UTC assay with NS has the potential to play a role in UC detection. The value of this assay still needs additional validation by large, multi‐center studies.

## Graphical abstract

Nanostructured Substrates (NS) could be used to enrich exfoliated nucleate cells from urine samples. The assay based on NS enrichment and immunocytology could be a novel non‐invasive diagnostic tool for urothelial carcinoma (UC).

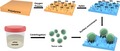

## 1 INTRODUCTION

In China, the morbidity (80.5%) and mortality (32.9%) of urothelial carcinoma (UC) have been increasing over the past few years.^1^ Early detection of UC is vital for the improvement of prognosis and survival.^2^ Cytology and ureterocystoscopy along with biopsy remain the fundamental means for clinical detection of UC. Ureterocystoscopy is not only invasive but also with a high miss rate, especially in UC in situ and upper tract UC.^3^ Seeking for diagnostic methods with higher accuracy in a noninvasive manner has been the goal of many investigators for a long time.

There are two main types of noninvasive methods for UC detection: one is based on the tumor antigens in urine, and the other is based on the exfoliated tumor cells. Noninvasive detection for UC tumor markers, such as ELISA analysis of nuclear matrix protein 22 (NMP22) and/or bladder tumor antigen (BTA),^4^, ^5^ have been clinically implemented. Nevertheless, low sensitivity and high false positives limit their relevant clinical use.^6^ Differing from detecting tumor markers, cytology is a noninvasive and low‐cost approach based on cell morphology and can provide 89%‐100% diagnostic specificity. Cytology is the most frequently used noninvasive diagnostic method in clinical setting, and has been considered as a routine examination in initial diagnosis and follow‐up of UC. However, it has a nonnegligible inherent shortcoming, low sensitivity (8.9%‐65%).^7^, ^8^ To address this issue, immunocytology was developed, in which fluorescence‐labeled antibodies are used to stain tumor‐specific antigens derived from exfoliated cells for identifying cancer cells.^9^ ImmunoCyt (Scimedx), a commercial product approved by the Food and Drug Administration of United States, offers sensitivity of 45% and specificity of 52%, respectively, using multiple antibodies targeting cytoplasmic mucins and carcinoembryonic antigens.^10^ Recently, by adopting antibodies of p53, ki67, and cytokeratin (CK), the detection sensitivity and specificity increased to 68.9%‐91.1% and 74.3%‐97.5%, respectively.^11^, ^12^ Of note, the wide range of sensitivity provided by these commercial kits indicates that the high level of false negatives still exist. UroVysion is another cell‐based test for UC, which is a multiprobe fluorescence in situ hybridization assay designed to target aneuploidy of chromosomes 3, 7, 17, and loss of 9p21. UroVysion can be used as a supplement to cytology for detecting high‐grade UC with good sensitivity, but still unsatisfactory for the detection of low‐grade UC.^13^ UroVysion also adds cost burden in the long‐term follow‐up.

Nanostructured substrates (NS) have been successfully used for detecting rare circulating tumor cells (CTCs).^14^ The major advantage of NS is the ultrahigh detection sensitivity. Over 95% of CTCs can be efficiently detected among the millions of white blood cells (WBCs).^15^ Inspired by this, we developed polystyrene‐based NS for efficient enrichment of exfoliated cells in urine followed by the identification of potential cancer cells with immunoassay. In this study, we optimized the parameters for NS preparation, evaluated the cell enrichment efficiency, and selected antibodies for specific identification of urinary tumor cells (UTCs). The aim of this study was to evaluate the diagnostic ability of our method by comparing it with traditional cytology, and apply it to detect UTCs from UC patients before and after surgery.

## 2 MATERIALS AND METHODS

### 2.1 NS preparing

**Figure 1:**
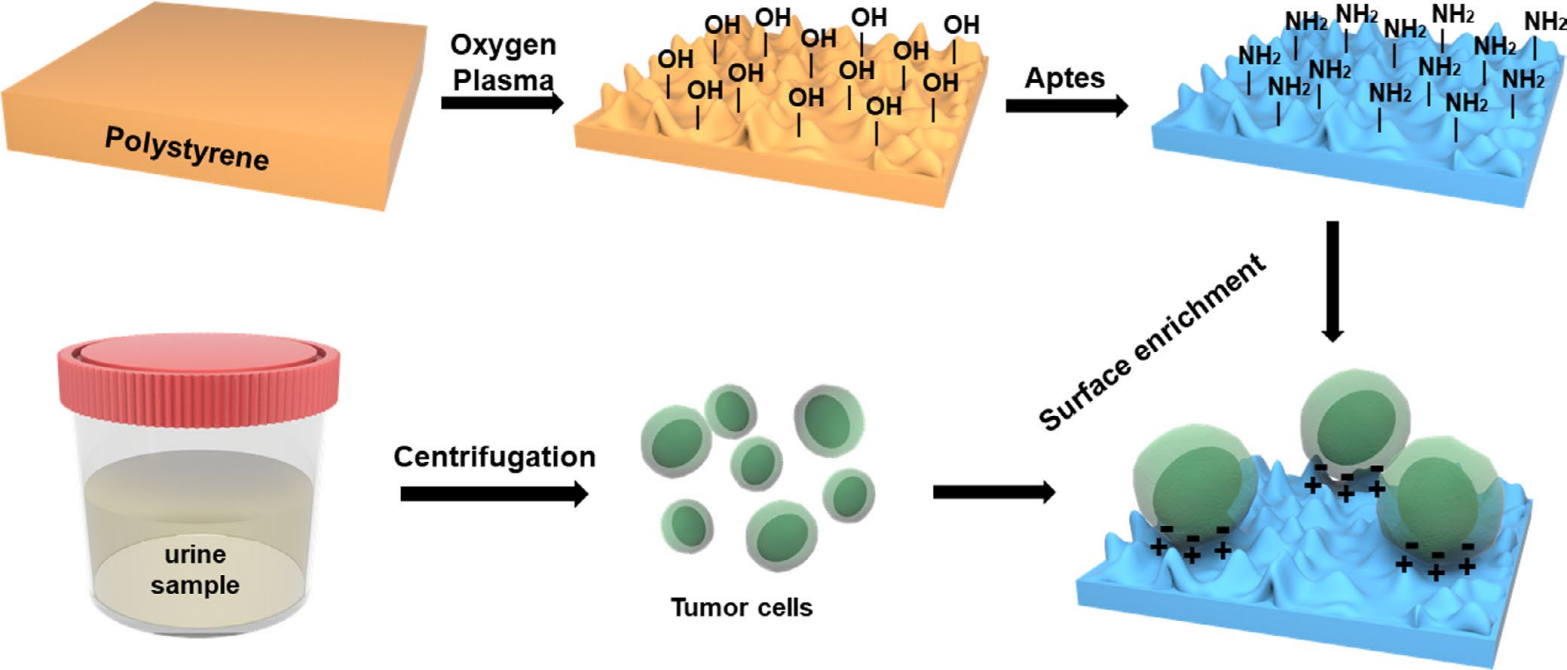
Schematic diagram of amine‐functionalized NS preparation and urinary tumor cells enrichment. Tumor cell membranes are negatively charged; the amine‐functionalized NS is positively charged. Aptes = 3‐minopropyltriethoxysilane; NS = nanostructured substrates

To prepare the unique NS for our study, corning 60‐mm polystyrene (PS) dishes were exposed to oxygen plasma according to the previous publications.^15^ Then the substrates were amine‐functionalized by immersing in 3% 3‐minopropyltriethoxysilane (APTES) in ethanol for 1 hour at room temperature (Figure 1). After that, the substrates were washed by deionized water for three times and cured at 80°C for 1 hour.

Atomic force microscope (AFM) was used to evaluate the surface topography of NS. Using a Kratos AXIS Ultra DLD X‐ray photoelectron spectrometer, the characteristic of NS was investigated by X‐ray photoelectron spectroscopy (XPS) under a hemispherical analyzer and a monochromatic Al*K_α_* X‐ray source using 20‐eV pass energy with 0.3 eV in high‐resolution measurement. The photoelectron takeoff angle was adjusted to 90° to collect signal. The surface charging effects was compensated by a C 1s hydrocarbon carbon peak at 281.0 eV to reference binding energies.

### 2.2 Cell culture and cell binding yields

Human bladder cancer cell lines (T24 and 5637) and normal ureteral epithelial cells (SV‐HUC‐1) were cultured in cell incubator by RPMI‐1640 supplemented with 10% fetal bovine at 37°C maintaining 5% CO_2_.

For cell seeding or spiking, T24 cells were separated by trypsinization and counted by hemocytometer with trypan blue staining. To find the appropriate incubation time, 100 000 cells were added to the amine‐functionalized NS and untreated PS for 30 minutes, 1 hour, 2 hours, 4 hours, and 6 hours. To detect the cell binding yields, T24 cells were diluted to 50, 5 × 10^2^, 5 × 10^3^, 5 × 10^4^, and 5 × 10^5^ cells/mL and added to NS. Moreover, to investigate the capture yields of tumor cells in urine, we spiked 50, 100, and 200 T24 cells into 50‐mL cell free urine samples, respectively, and verified the binding yields of NS (Figure 1).

### 2.3 Population and samples

From January 2018 to December 2018, 130 patients with suspicious bladder tumor and another 50 normal controls were enrolled in this study (Table 1). Written consent was obtained from all participants in accordance with the Declaration of Helsinki, and all protocols concerning the use of human samples in this study were approved by the Ethics Committee of the Nanjing Drum Tower Hospital. Transurethral resection was performed for the patients with suspicious tumor. Histopathologic classification was performed according to the World Health Organization (WHO)/International Society of Urologic Pathology 2014 consensus classification. Histologic evaluation of tissue samples obtained from biopsy specimens or surgical resections served as the “gold standard.” The urine samples for both cytology and UTC assay were collected before biopsy, surgery, or any other manipulation. Postoperative urine samples were collected at least 3 weeks after surgery. About 30‐mL morning urine was collected for each urine cytology, and another 100‐mL morning urine was collected for UTC assay.

**Table 1:** Clinical and histopathological characteristics

**Urothelial carcinoma patients**		
Gender	N	%
Male	78	72.9
Female	29	27.1
Age	Mean (year)	Range (year)
	66.5	42‐87
Grade	N	%
Low‐grade	37	34.6
High‐grade	70	65.4
Invasive	N	%
Yes	70	65.4
No	35	32.7
Unknown	2	1.9
Total (UC)	107	100

**Nonurothelial carcinoma patients**		
Gender	N	%
Male	46	63.0
Female	27	37.0
Age	Mean (year)	Range (year)
	59	29‐90
Urological pathology	N	%
IPB	6	8.2
BPH	3	4.1
Cystitis glandular	12	16.4
Other	2	2.7
Nonurological disease^a^	50	68.5
Total (non‐UC)	73	100
Total	180	100

Abbreviations: UC, urothelial carcinoma; IPB, inverted papilloma of the bladder; BPH, benign prostatic hyperplasia.

a Participants whose urinary system examination is completely normal.

### 2.4 Cytology

Specimens were processed for liquid‐based cytology (Thin‐Prep; Cytyc, Marlborough, Mass) and stained according to the standard Papanicolaou procedure. The cytology categories were defined by skilled pathologists in the department of our hospital with blinding to our study, and the diagnostic criteria of the Paris System were referred.^16^ Urine cytology tests for each patient were once a day for three consecutive days. Cytology was defined “positive” when one or more of the three tests reached positive standard. The “positive” category included low‐grade urothelial carcinoma (LUC) and high‐grade urothelial carcinoma (HUC). “Atypia results” including ‘‘suspicious’’ and ‘‘atypical’’ were assigned to the “negative” category.^17^

### 2.5 UTC assay

Specimens were stored at 4°C and centrifuged at 200 g for 5 minutes within 1 hour of urine sample collection. Pellets were resuspended by 3‐mL FPBS (PBS containing 2% fetal calf serum) and transferred to sample storage tubes. These tubes were sent to the laboratory with ice bags on the same day and received the next day. The whole process took about 24 hours.

Once received, urine specimens were handled immediately. They were centrifuged at 200 g for 5 minutes after which 1‐mL FPBS was used to resuspend cells. For the hematuria samples, the depletion of red cells by 1 X Lysis Buffer was necessary. Cells were added to the prepared NS, then cultured at 37°C for 1 hour followed by 4°C for 10 minutes. After the fluid removed, cells were treated with 1 mL 4% Paraformaldehyde at 4°C for 10 minutes. After that, precooling methanol was used to treat cells for 10 minutes at −20°C. Cells were washed by PBS three times and blocked by 5% nonfat milk for 30 minutes at room temperature afterwards.

Cells were immunostained with CK20 (eBioscience) or EpCAM (Abcam), CD45 (eBioscience), and CD11b (Abcam). Nuclei were stained with DAPI (Sigma). CK20 was used as the main marker to detect UC cells that exfoliated in voided urine. CD45 and CD11b were used to mark WBCs and myeloid derivatives, respectively. Cells with DAPI^+^CK20^+^CD45^−^CD11b^−^ were identified as tumor cells. Cytell Cell Imaging System (GE Lifesciences) was used to high‐throughput capture the stained samples. The supporting software was used to analyze and count cells.

### 2.6 Statistics

GraphPad Prism v6.0 was used for the analysis, and the results were presented as mean ± SD. ROC analysis, Kappa analysis, and McNemar test were performed by SPSS 24.0. The diagnostic accuracy of UTC assay was evaluated by constructing a ROC curve and calculating AUC‐ROC. The difference from 0.5 reached statistical significance was calculated as the *P* value less than .05 would be identified as significant. Youden index = sensitivity + specificity − 1. The cutoff value was obtained when Youden index reached maximum. Kappa test was used to compare the consistency of cytology/UTC assay and pathology. A kappa value more than 0.6 indicates good agreement and a kappa value less than 0.4 indicates poor agreement. Sensitivity and specificity for cytology and UTC assay were calculated using the 2 × 2 contingency table. McNemar test was used to analyze the statistical differences in sensitivity and specificity of cytology and UTC assay.^18^ A value of *P *less than .05 was considered statistically significant.

## 3 RESULTS

**Figure 2:**
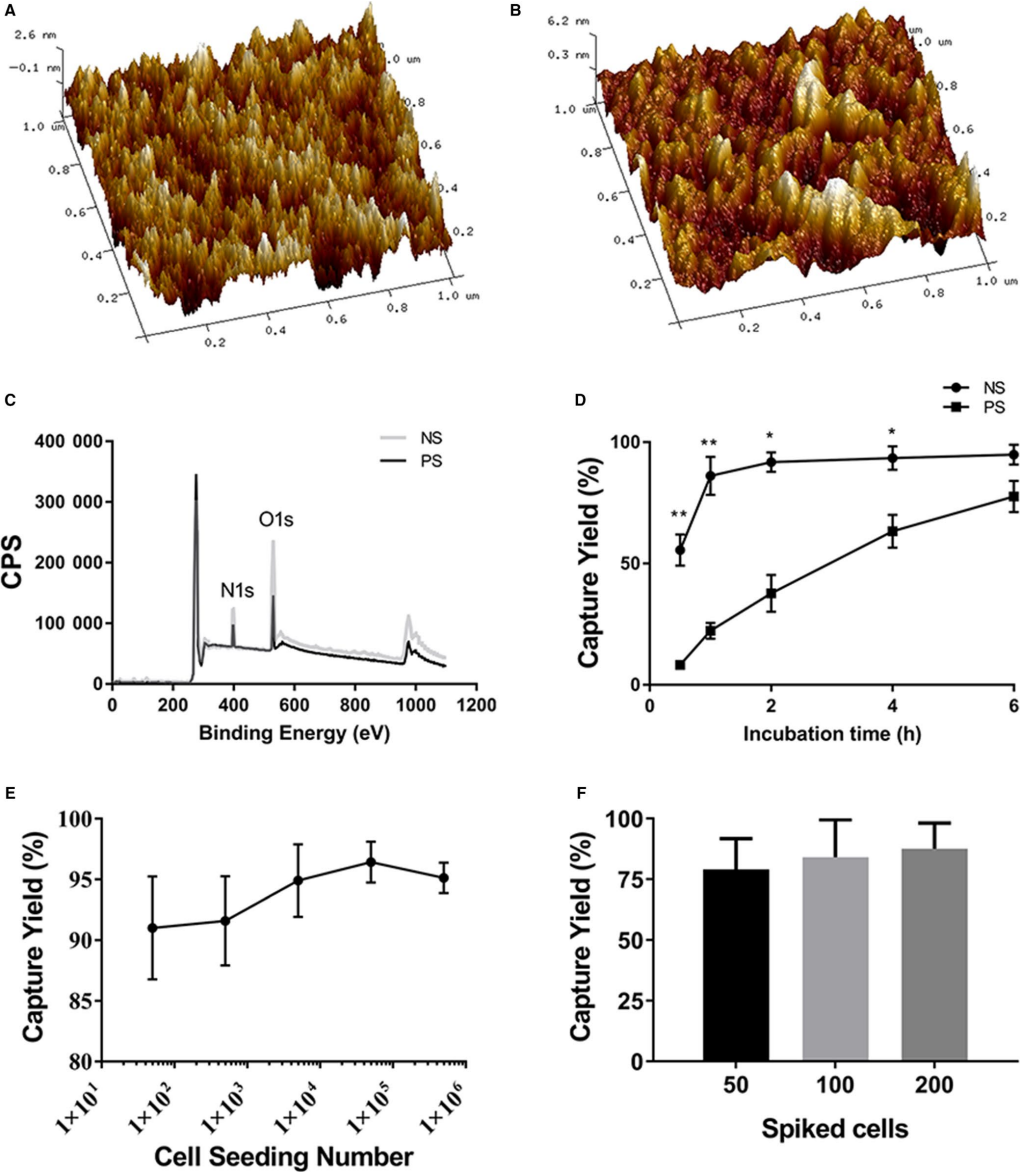
Performance characterization of amine‐functionalized NS. (A, B) Representative AFM images (1 × 1μm) of (A) untreated PS and (B) amine‐functionalized NS (PS treated with oxygen plasma and amine‐functionalized by APTES). The roughness of NS is significantly improved compared to PS; (C) XPS elemental analysis of PS (black line) and NS (gray line) samples; (D) Binding yields as a function of incubation time for T24 cells on PS and NS; (E) Binding yields at 1 h as a function of seeding density for T24 cells on NS; (F) Capture yields of T24 cells spiked in urine samples at different concentrations (50, 100, 200 cells spiked in 50‐ml urine, respectively) on NS 1 h after cell seeding. NS = nanostructured substrates; PS = polystyrene; APTES = 3‐aminopropyltriethoxysilane; AFM = atomic force microscope; XPS = X‐ray photoelectron spectroscopy

We prepared the NS and detected its relative characteristics. First, AFM results showed the roughness of NS (Rq = 1.381 ± 0.2694, Ra = 1.119 ± 0.1081) (Figure 2B) was significantly higher than PS (Rq = 0.927 ± 0.1956, Ra = 0.7255 ± 0.1422) (Figure 2A) (Rq, *P* = .0342; Ra, *P* = .0045). XPS elemental analysis of NS showed a significant increase in the oxygen and nitrogen percentage, which confirmed the expected modification (Figure 2C). Then we compared the cell capture yields of NS and PS. We incubated the cells on NS and PS for 30 minutes, 1 hour, 2 hours, 4 hours, and 6 hours. At the time point of 1 hour, approximate 90% tumor cells were captured by NS, whereas only 22.3% by PS (capture yields: NS 86.159 ± 7.904% vs PS 22.276 ± 3.243%, *P* = .0088) (Figure 2D). We further characterized the binding yields by seeding the different numbers of T24 cells on NS. An average binding yields of 93.81 ± 2.374% was achieved when incubating for 1 hour (Figure 2E). Spiking different number of tumor cells into equal amount of cell free urine, we found an average binding yield of 83.5% (Figure 2F).

**Figure 3:**
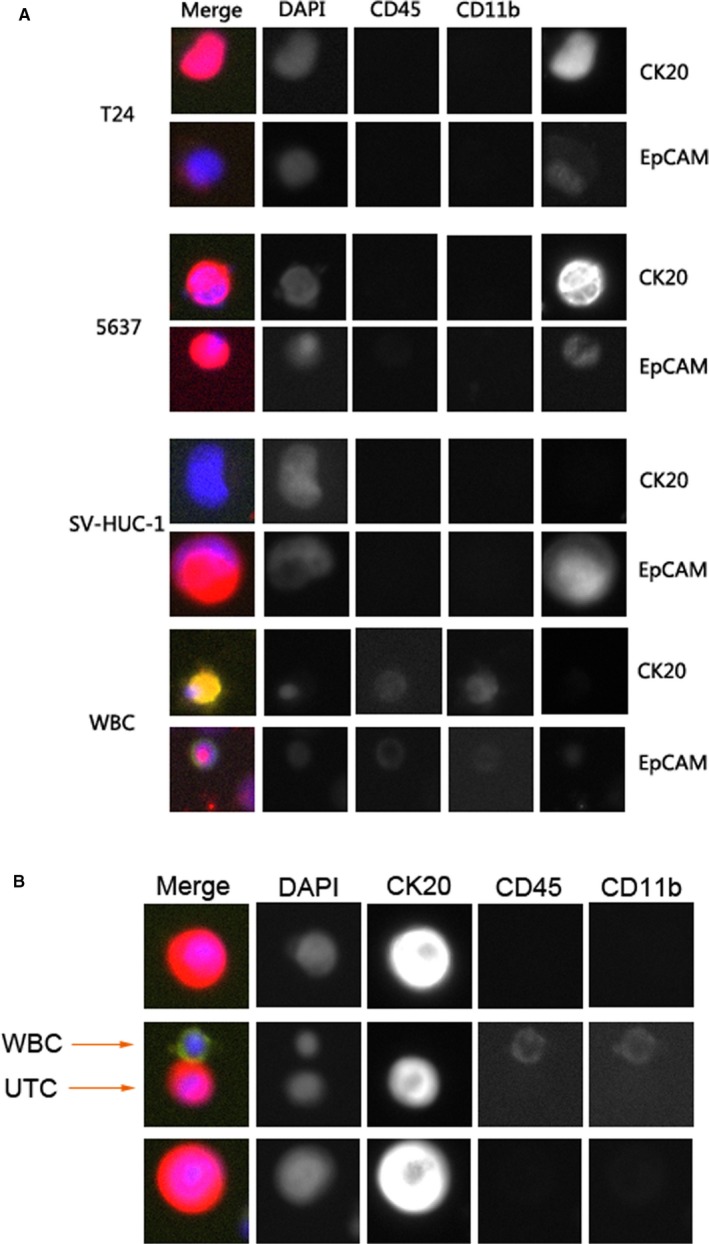
Efficacy of CK20/CD45/CD11b for discriminating cancer cells from healthy urothelial cells and WBCs captured by NS. CK20 was used as the main marker to detect urothelial cancer cells. CD45 and CD11b were used to mark WBCs and myeloid derivatives, respectively. (A) Graph showing the comparison of CK20 and EpCAM. The immunofluorescence tests were conducted on cell lines (T24, 5637, SV‐HUC‐1) and WBCs. Each cell line was divided into two parts, one part was for CK20 experiment and the other was for EpCAM experiment. CD45 and CD11b were tested in both CK20 experiment and EpCAM experiment. CK20 showed good discriminatory ability. T24 and 5637 cells were CK20^+^CD45^−^CD11b^−^, SV‐HUC‐1 cells were CK20^−^CD45^−^CD11b^−^ and WBCs were CK20^−^CD45^+^CD11b^+^. The combination of CK20, CD45, and CD11b demonstrated excellent ability to identify urothelial cancer cells. (B) Graph showing CK20/CD45/CD11b staining of UTCs and WBCs in urine sample isolated on NS. In the preliminary study, we validated the efficacy of CK20/CD45/CD11b marker combination in a large number of urine samples from bladder cancer patients. Here we show a representative example of such experiments, suggesting good ability of UTC assay in detecting UTCs in urine. UTC = urinary tumor cell

Then we used NS as the platform for immunofluorescence on urine exfoliated nucleated cells. We chose T24, 5637, SV‐HUC‐1 cell lines, and WBCs to test the antibodies for identifying urothelial tumor cells, including CK20/EpCAM, CD45, and CD11b (Figure 3A). Among them, SV‐HUC‐1 was the uroepithelium cell line served as a normal control. WBCs were collected from the peripheral blood of normal person with red cells lysed. T24 and 5637 cells were CK20^+^CD45^−^CD11b^−^ and EpCAM^+^CD45^−^CD11b^−^. SV‐HUC‐1 cells showed CK20^−^CD45^−^CD11b^−^ and EpCAM^+^CD45^−^CD11b^−^. WBCs were CK20^−^CD45^+^CD11b^+^ and EpCAM^+^CD45^+^CD11b^+^. Consequently, these results demonstrated that CK20, CD45, and CD11b were ideal choice for urothelial tumor cell detection. CK20^+^CD45^−^CD11b^−^ cells were considered positive. This marker combination was also verified in urine samples of bladder cancer patients, which showed a good effect on distinguishing tumor cells from WBCs (Figure 3B).

**Figure 4:**
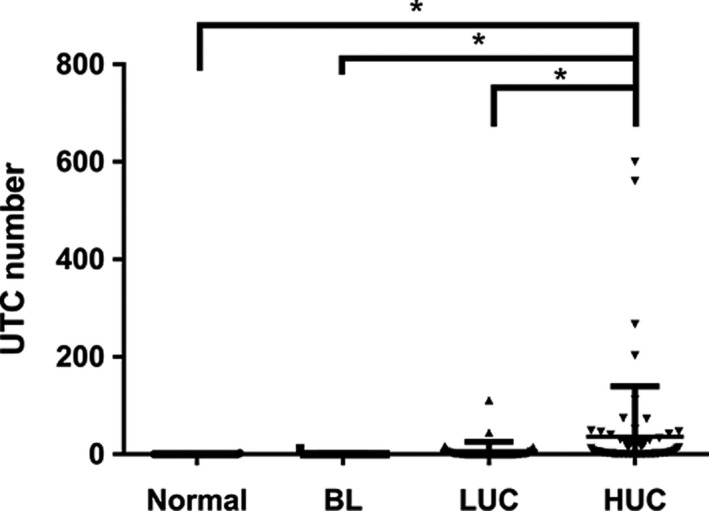
UTC number of the collected clinical urine samples stratified according to pathological outcomes. BL = benign lesion; LUC = low‐grade urothelial carcinoma; HUC = high‐grade urothelial carcinoma; UTC = urinary tumor cell. * represents *P < *.05

Urine samples of 180 participants (mean age: 61.5 years; range: 21‐90 years) were prospectively collected for analysis. The final pathological results of the participants with bladder tumor were outlined in Table 1. Among them, 23 patients were confirmed with benign lesions (BL), 37 were with LUC, and 70 were with HUC. We looked through the UTC number in four subgroups classified by pathology, and found that the UTC number of HUC group (44.36 ± 102.9) was significantly larger than normal group (0.05882 ± 0.343, *P* = .0139), BL group (0.3478 ± 1.668, *P* = .0439), and LUC group (6.541 ± 19.37, *P* = .0293) (Figure 4).

ROC curve analysis and Youden index were used to assess the performance and define a suitable cutoff value of UTC number for UC diagnosis (Table 2). The AUC of UTC assay was greater than 0.8 (AUC 0.888, *P* < .001) which indicated good diagnostic performance. Youden index was used to find the cutoff value. It was found that the cutoff values of UTC number in different subgroups were the same (0.5 per sample) regardless of grade and stage. For the ease of use, we finally chose UTC number ≥ 1 cell per sample as the threshold of positive result.

**Table 2:** Determining cutoff value of UTC assay by ROC curve analysis and Youden index

	AUC	*P* value	95% CI	Youden index	Cutoff value
Total UC	0.889	<.001	0.839 to 0.939	0.777	0.5
Grade					
Low	0.836	<.001	0.742 to 0.929	0.676	0.5
High	0.917	<.001	0.865 to 0.969	0.83	0.5
Invasiveness					
Yes	0.875	<.001	0.789 to 0.961	0.744	0.5
No	0.893	<.001	0.834 to 0.952	0.787	0.5

Abbreviations: 95% CI, 95% confidence interval; AUC, area under curve; ROC, receiver operating characteristic; UC, urothelial carcinoma; UTC, urinary tumor cell.

**Figure 5:**
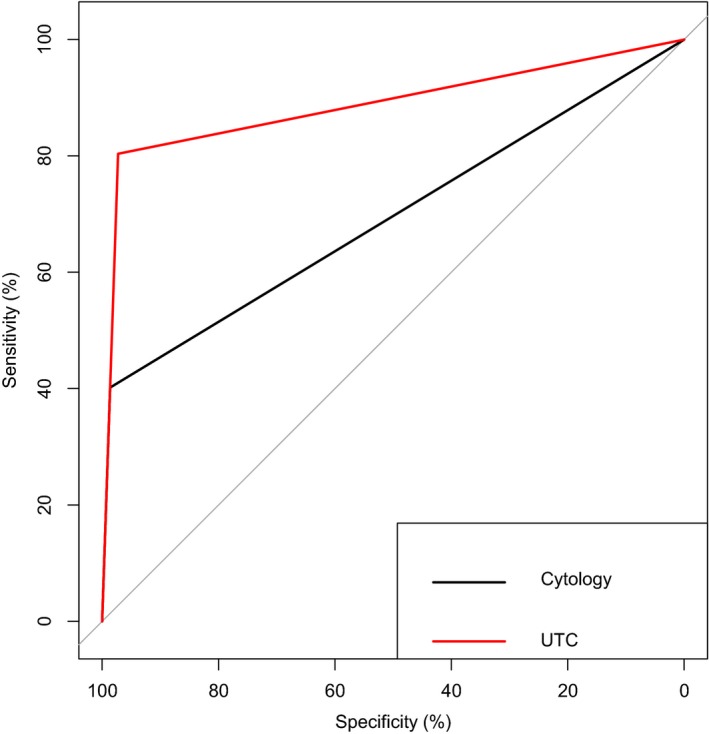
ROC curves for UTC assay and cytology. AUC for UTC assay (0.888) was significantly greater than that for cytology (0.694, *P* < .001). AUC = area under curve; ROC = receiver operating characteristic

Next, we compared the diagnostic efficacy between UTC assay and cytology. ROC curve analysis showed UTC assay had the AUC of 0.888 (*P* < .001) while cytology had the AUC of 0.694 (*P* < .001) in UC detection (Figure 5). In different subgroups (low/high‐grade UC or invasive/noninvasive UC), ROC curve analysis also revealed that UTC assay had better performance than cytology (see in Table S1). The sensitivities, specificity, and Kappa values of cytology, UTC assay, and UTC assay combined with cytology were detailed in Table 3. McNemar test was used to compare the sensitivities and specificities between cytology and UTC assay. Overall, the sensitivity of UTC assay was significantly superior to cytology (80.4% vs 40.2%, *P* < .05). Of note, in subgroup analysis, UTC assay had a much better performance for low‐grade group than cytology (70.27% vs 18.92%, *P* < .05). Meanwhile, the specificity of cytology (98.63%) and UTC assay (97.26%) was similar (*P* = 1). Furthermore, Kappa value showed UTC assay had a good agreement with pathological diagnosis while cytology had a poor agreement (0.746 vs 0.341, *P* < .001). UTC assay combined with cytology showed a sensitivity of 84.1%, a specificity of 95.9% for the diagnosis of UC.

**Table 3:** Diagnostic performance of cytology, UTC assay and the combined use of cytology and UTC assay

	Cytology	UTC	Cytology and UTC
Sensitivity			
Total UC	40.2%	80.4%	84.1%
Low‐grade	18.9%	70.3%	75.7%
High‐grade	51.4%	85.7%	88.6%
Specificity			
Total UC	98.6%	97.3%	95.9%
McNemar test of sensitivity			
Total UC	<0.001		—
Low‐grade	<0.001		—
High‐grade	<0.001		—
Kappa value (*P* value)			
Total UC	0.341 (<0.001)	0.746 (<0.001)	0.776 (<0.001)
Low‐grade	0.218 (0.004)	0.718 (<0.001)	0.745 (<0.001)
High‐grade	0.505 (<0.001)	0.832 (<0.001)	0.846 (<0.001)

Abbreviations: UC, urothelial carcinoma; UTC, urinary tumor cell.

Finally, we compared the UTC number of UC patients (n = 20) before and after surgery (Table 4). Twenty of 20 patients were UTC positive before surgery and 5 of 20 patients were UTC positive after surgery. We found that the UTC numbers significantly decreased 4 weeks after surgery during follow‐up (*P* < .001).

**Table 4:** Individual UTC assay outcomes at follow‐up postoperatively in comparison with preoperative outcomes in 20 patients

Case	Gender	Age (year)	Diagnosis	Stage^a^	UTC number before surgery	UTC number after surgery
#1	Male	53	Low‐grade UC, focal high‐grade UC	T1	5	0
#2	Male	47	High‐grade UC	T1	2	1
#3	Male	55	High‐grade UC	T1	25	1
#4	Male	74	High‐grade UC	Ta	3	1
#5	Male	85	Low‐grade UC, focal high‐grade UC	Ta	44	0
#6	Female	70	Low‐grade UC	Ta	2	0
#7	Male	65	Low‐grade UC, focal high‐grade UC	T3	5	0
#8	Female	70	Low‐grade UC	Ta	7	0
#9	Male	53	Low‐grade UC	T1	3	0
#10	Male	60	Low‐grade UC	Ta	2	0
#11	Male	56	Low‐grade UC	Ta	3	0
#12	Male	81	High‐grade UC	T1	49	0
#13	Male	55	High‐grade UC	T1	25	0
#14	Female	75	High‐grade UC	T1	40	0
#15	Male	66	High‐grade UC	T1	6	0
#16	Male	80	High‐grade UC	T4	5	4
#17	Male	46	High‐grade UC	T1	1	0
#18	Male	82	High‐grade UC	T2	73	0
#19	Male	69	Low‐grade UC	Ta	2	0
#20	Female	85	High‐grade UC	T1	29	1

Abbreviations: UC, urothelial carcinoma; UTC, urinary tumor cell.

a Some of these 20 patients underwent radical cystectomy, and the stage was dependent on the final pathology.

## 4 DISCUSSION

It is reported that NS could be used as an ultrasensitive tool for enriching rare CTCs from blood. Meanwhile, NS could keep most captured cells viable for molecular analysis. In the present study, we established a method for the detection of UTCs by immunofluorescence assay on NS. We confirmed that NS can efficiently absorb nucleated cells from urine and work well as the platform for immunofluorescence assay. The diagnostic efficacy of UTC assay was superior over cytology in UC. Of note, UTC assay had a better sensitivity in low‐grade subgroup (70.27%) and the overall specificity (97.26%) was similar to cytology.

Urine cytology is the most common noninvasive diagnostic and monitoring modality for bladder cancer and frequently used as an adjunct to cystoscopy. Although with high specificity (>90%), it often leads to equivocal diagnosis in LUC due to subtle cytological feature and low cell spilling.^19^ The UroVysion assay is a commercially available test detecting specific chromosome abnormalities for UC diagnosis. Overall sensitivity and specificity of the UroVysion assay were reported to be 72% and 83%, respectively.^20^ Like cytology, UroVysion has been shown to have a lower sensitivity for the detection of LUC, as compared with HUC.^21^ Dimashkieh et al evaluated the sensitivity and specificity of UroVysion and cytology in the same urine sample, and UroVysion revealed overall sensitivity and specificity of 61.9% and 89.7%, respectively; and 40.8% and 87.8% respectively for LUC.^13^ Besides, UroVysion is technically complex and relatively expensive. Immunocytology is an “enhanced version” of cytology. ImmunoCyt is an immunocytologic test established more than 20 years ago, using three monoclonal antibodies (19A211, M344, and LDQ10) to detect the tumor‐specific antigens of urothelial tumor cells. The 19A211 antibody recognizes a high‐molecular weight form of glycosylated carcinoembryonic antigen (CEA). The other two antibodies LDQ10 and M344 detect mucin antigens. Previous studies have demonstrated a high sensitivity of ImmunoCyt in detecting UC, and improved the performance for LUC compared with cytology.^22^, ^23^, ^24^ Sullivan et al evaluated the utility of ImmunoCyt, UroVysion, and urine cytology in a “Split‐Sample” study, demonstrating that ImmunoCyt is more sensitive than either cytology or UroVysion in detecting low‐grade tumors.^23^ Although with better sensitivity for UC detection, the lower specificity (61%‐78%) of ImmunoCyt compared with cytology or UroVysion limited its wide application. Additionally, ImmunoCyt has great variation in diagnosis accuracy and is not approved as a stand‐alone test for UC.^25^ For these reasons, we did not include this assay in our study. The factors affecting the efficiency of immunocytology mainly include the following three aspects: the amount of collected exfoliated tumor cells, tumor‐specific biomarkers, and diagnostic differences between cytopathologists. Thus, optimizing the three aspects is expected to improve the efficiency of immunocytology.

In theory, maximizing the acquisition of nucleated cells in urine helps to increase the likelihood of UTCs detection. Nanostructure‐based cell enrichment is now widely used in the capture of CTCs from blood. Yuan et al showed that high‐power oxygen plasma‐treated PS had a surface with homogeneous nanoscale roughness, which unbiased enhance the binding of the nucleated cells.^15^ In the present study, we modified the high‐power oxygen plasma‐treated PS with APTES to enhance the cell adhesion. Meanwhile, the different sizes of PS dishes can be selected according to the amount of cells, so as to avoid the accumulation of cells which may affect the fluorescence detection. Our nanostructure‐based cell enrichment method can capture approximate 90% tumor cells in urine at the time point of 1h, which is much stronger than the untreated PS. Beside the efficient, strong, and rapid attachment of tumor cells in urine, the NS could retain morphology of the binding cells for further investigation, such as immunofluorescence. To our knowledge this study represents the first application of unbiased enrichment technology in urine. This novel enrichment method can be used more widely for detecting rare tumor cells falling off primary tumor mass into urine to improve early diagnosis.

The biological markers used in immunofluorescence are another critical factor affecting the diagnostic accuracy of immunocytology. At present, the relevant markers are mainly divided into two categories. The first category discriminates between benign and malignant cells on the whole, such as CK20 and some cyclins (P53 and MCM‐2).^26^, ^27^ Among them, CK20 is a high molecular weight CK normally expressed in umbrella cells, and also expressed in the deep layers of the urothelium in the presence of carcinogenesis. Therefore, nonumbrella CK20^+^ cells appearing in urine can be used as an indicator for judging the presence or absence of UC, with a diagnostic sensitivity of 70%‐82% and a specificity of 71%‐78%.^28^, ^29^ Another important advantage of CK20 is its high diagnostic efficiency for low‐grade tumors. A study of Wadhwa et al in 2017 showed that the CK20 test reduced the rate of undetectable exfoliated cells in patients with LUC from 57.1% to 10.7%.^24^ Those are why we choose CK20 as the marker for UC in this study. The second category of markers is associated with the degree of malignancy of the tumor and can be used to determine the grade and prognosis of bladder cancer, such as P16^INK4a^ and CXCR4.^30^, ^31^ Although not included in the present study, the application of these biomarkers is expected to improve the efficiency of diagnosis, and can make a preliminary judgment on the classification and prognosis of tumors. It could be a new direction for our further research. The majority of WBCs highly express CD45, which is a WBC marker. However, some WBCs have low or absent expression of CD45 such as neutrophils, myeloid‐derived suppressor cells (MDSCs), or other immature myeloid subsets.^32^ To identify these CD45^low/−^ WBCs, we chose CD11b, a myeloid‐specific marker which is highly expressed at an early stage of myeloid development and strongly expressed on CD45^low^ myeloid derivatives, including both MDSCs and neutrophils.^33^ More importantly, some mature myeloid derivatives like neutrophils, which express the low levels of CD45 increase in patients with progressive cancer, and stain positive for CK. CD11b^+^CD45^low^ cells were reported to have strong pCK staining and may be misidentified as CTCs based on CK staining.^34^ Similarly, false positive may exist for the definition of UTCs in our study if based solely on CK20 and CD45. After we added CD11b, the CK20^+^CD45^low^ cells can be divided into true UTCs (CK20^+^CD45^low^CD11b^−^) and false‐positive myeloid derivatives (CK20^+^CD45^low^CD11b^+^), thus improving the specificity of the UTC assay.

Another new technology that can improve the efficiency of immunocytology is the application of fully automated high‐resolution cell imaging systems, such as Cytell system and Operetta system. These systems combine the functions of a high‐resolution digital microscope, an image cell analyzer, and a cell counter. They allow for maximum analysis of fluorescent staining results and high‐throughput analysis of cell structure. That is to say, besides the immunofluorescence information, morphologic information (eg, nuclear size) can also be obtained, which is theoretically an analogy of getting parameters of cytology to some extent. Since studies have shown that combining immunocytology with urine cytology can increase the diagnostic sensitivity,^35^ the imaging system may help to elevate the sensitivity of UTC assay. Indeed, we found that the combined use of UTC assay and cytology has an increased diagnostic sensitivity (84.1%) and a slightly lower specificity (95.9%). However, the diagnostic value of cell morphologic information obtained by high‐resolution cell imaging systems needs to be furtherly validated. As cytology and ImmunoCyt both blot cells on the microscope slides, observers need to read the slides by microscope examination fields by fields, which is time‐consuming and malignant cells may be missed. Automated cell imaging systems could overcome these disadvantages. Comparing with cytology, it only needed to exclude the images without cell morphology which required little cytopathology experience and had high interobserver reproducibility. At the same time, the automatic recognition and interpretation function of the computer can significantly reduce the workload of pathologists. The actual operation and observation time for UTCs detection would be less than 10 minutes.

The total number of UTCs can roughly judge tumor property, which is consistent with the characteristics of UC, as tumor cells are more prone to shed with the increase in tumor grade and stage. Setting a suitable cutoff threshold can transform UTC number into a dichotomous variable and standardize the interpretation of UTC assay. In spite of the large range of cell numbers in urine samples (from 24 to 535 106 cells), we found that UTC number had the same cutoff value in LUC and HUC. So we finally set ≥ 1 cell per sample as UTC assay positive, making it simple and convenient. With the new standard, we compared the diagnostic capability of UTC assay and cytology in UC. The overall sensitivity of UTC assay in the identification of suspected malignancy was 80.4% and the sensitivity was notable in LUC (70.27%). The specificity of UTC assay was comparable to cytology (97.26% vs 98.63%, *P* = 1). Higher sensitivity and unaffected specificity suggests its potential utility as an adjunct biomarker in LUC.

The surveillance of nonmuscle invasive bladder cancer mainly relies on routine cystoscopy and cytology. Cytology has a low sensitivity especially for low‐grade tumors, whereas cystoscopy remains an invasive examination. More and more urine‐based biomarkers have been developed for the follow‐up of bladder UC, such as NMP22, BTA, ImmunoCyt, and UroVysion. However, NMP22 had a poor sensitivity level for detecting recurrence.^36^ BTA tests had low sensitivity and high rates of false‐positive results.^37^ For ImmunoCyt, interobserver variability has been found to be a major drawback.^38^ The sensitivity of UroVysion varied, and was recommended specifically for the setting of atypical cytology or cystoscopy.^39^ In the present study, the UTC numbers before and after surgery were initially compared in a group of patients. We set the retest time point of more than 4 weeks due to possible residual tumor cells may stay shortly after surgery. The vast majority of UTC numbers decreased significantly after surgery, yet 5 of 20 still had positive results, suggesting there might be residual tumor cells. Regular intravesical chemotherapy and close monitoring are necessary for the five patients. Further study is warranted to determine the value of UTC assay in surveillance.

Degeneration and autolysis of cells is a problem needed to be solved. Urine is not a suitable environment for cell survival due to the pH value, limited nutrition and bacterial contamination. The number of cells in urine sample will reduce about 50% every single day, even if stored in 4°C (data not shown). So all the urine samples here were proceeded within 1 hour after collection in order to maintain cell morphology and activity. Specific management can achieve long‐term storage of urine, keeping metabolite stability and sample integrity even at room temperature.^40^, ^41^, ^42^ In our future work, we will look for suitable preservation techniques to improve sample processing, and assess the possible effects of long‐term storage on the viability of exfoliated urinary cells and immunofluorescence assay. In addition, we need to establish standardized sample collection, transportation, and inspection processes for further commercial application.

## 5 CONCLUSION

From the results of our study, NS could be used for the enrichment of tumor cells in urine samples and engaged as a platform for immunofluorescence assay. UTC assay with NS may be a novel noninvasive diagnostic tool for UC, but the value of this assay still needs additional validation by large, multi‐center studies.

## CONFLICT OF INTEREST DISCLOSURES

None.

## AUTHOR CONTRIBUTIONS

Xin Wang and Shiwei Zhang were involved in investigation, methodology, data acquisition, and writing‐original draft. Yuanyuan Gu was involved in data acquisition, statistical analyses, and writing‐original draft. Rong Yang was involved in design and methodology of the study, study supervision, and editing. Hongqian Guo and Feng Fang were involved in study supervision and administrative support. Gangqiang Li and Qun Zhang were involved in study design and methodology and study supervision. Tianyao Liu, Tianwei Wang, Haixiang Qin, and Bo Jiang were involved in experimental support, investigation. Lin Zhu, Yajun Li, Haozhi Lei, and Ming Li were involved in project administration and statistical analyses. All the authors reviewed and accepted the final version of the article.
