# Supplementary material for: Unbiased enrichment of urine exfoliated cells on nanostructured substrates for sensitive detection of urothelial tumor cells
Source: Cancer Med. 2019 Nov 10;9(1):290–301. doi: 10.1002/cam4.2655 (PMC6943141; doi:10.1002/cam4.2655)
Supplement: Supplementary file 1 [file CAM4-9-290-s001.docx]

**Supplementary Table 1.** ROC curve analysis of UTC and cytology in subgroups divided on the basis of pathology.

|  | | Cytology | | | UTC assay | | |
| --- | --- | --- | --- | --- | --- | --- | --- |
|  | | AUC | *P* value | 95% CI | AUC | *P* value | 95% CI |
| Total UC | | 0.694 | <0.001 | 0.619 to 0.769 | 0.888 | <0.001 | 0.837 to 0.939 |
| Grade | Low | 0.588 | 0.134 | 0.470 to 0.706 | 0.838 | <0.001 | 0.744 to 0.931 |
|  | High | 0.750 | <0.001 | 0.668 to 0.833 | 0.915 | <0.001 | 0.862 to 0.968 |
| Invasiveness | Yes | 0.722 | <0.001 | 0.607 to 0.837 | 0.872 | <0.001 | 0.785 to 0.959 |
|  | No | 0.679 | <0.001 | 0.590 to 0.768 | 0.893 | <0.001 | 0.834 to 0.952 |

Abbreviations: ROC, receiver operating characteristic; UTC, urinary tumor cells; AUC, area under curve; UC, urothelial carcinoma; 95% CI, 95% confidence interval.
